# 2-Bromo-4-methyl­benzonitrile

**DOI:** 10.1107/S1600536809048983

**Published:** 2009-11-21

**Authors:** Muhammad Shahid, Munawar Ali Munawar, Sohail Nadeem, Waqar Nasir, Muhammad Salim

**Affiliations:** a Institute of Chemistry, University of the Punjab, New Campus, Lahore, Pakistan

## Abstract

The title mol­ecule, C_8_H_6_BrN, is almost planar (r.m.s. deviation for the non-H atoms = 0.008 Å). In the crystal, weak π–π stacking inter­actions [centroid–centroid separations = 3.782 (2) and 3.919 (2) Å] generate [100] columns of mol­ecules.

## Related literature

For the synthesis, see: Johnson & Sandborn (1941 ▶). 2-Bromo-4-methyl­benzonitrile derivatives are used as inter­mediates in the synthesis of phthalocyanine dyes. For applications of phthalocyanine dyes in photo redox reactions and photodynamic cancer therapy, see: Simon & Sirlin (1989 ▶); Simon *et al.* (1989 ▶).
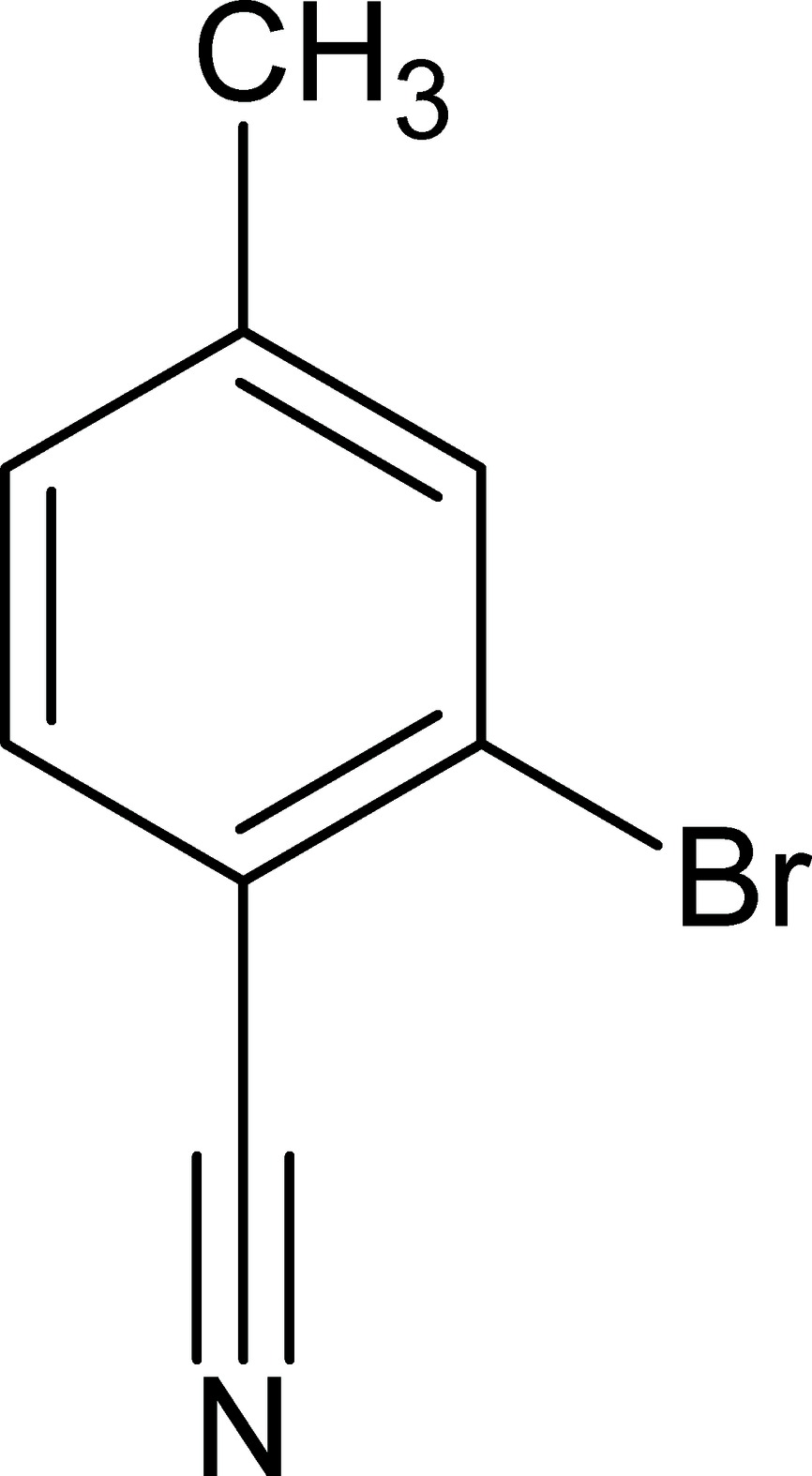


## Experimental

### 

#### Crystal data

C_8_H_6_BrN
*M* *_r_* = 196.05
Triclinic, 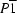

*a* = 7.5168 (11) Å
*b* = 7.8383 (11) Å
*c* = 7.9428 (11) Å
α = 69.243 (7)°
β = 64.375 (8)°
γ = 87.567 (8)°
*V* = 391.14 (10) Å^3^
*Z* = 2
Mo *K*α radiation
μ = 5.17 mm^−1^
*T* = 296 K
0.41 × 0.28 × 0.19 mm

#### Data collection

Bruker Kappa APEXII CCD diffractometer
Absorption correction: multi-scan (*SADABS*; Bruker, 2007 ▶) *T*_min_ = 0.226, *T*_max_ = 0.440
8084 measured reflections
1921 independent reflections
1244 reflections with *I* > 2σ(*I*)
*R*_int_ = 0.025

#### Refinement

*R*[*F* ^2^ > 2σ(*F* ^2^)] = 0.033
*wR*(*F* ^2^) = 0.084
*S* = 1.01
1921 reflections
92 parameters
H-atom parameters constrained
Δρ_max_ = 0.44 e Å^−3^
Δρ_min_ = −0.49 e Å^−3^

### 

Data collection: *APEX2* (Bruker, 2007 ▶); cell refinement: *SAINT* (Bruker, 2007 ▶); data reduction: *SAINT*; program(s) used to solve structure: *SHELXS97* (Sheldrick, 2008 ▶); program(s) used to refine structure: *SHELXL97* (Sheldrick, 2008 ▶); molecular graphics: *ORTEP-3* (Farrugia, 1997 ▶) and *PLATON* (Spek, 2009 ▶); software used to prepare material for publication: *WinGX* (Farrugia, 1999 ▶) and *PLATON*.

## Supplementary Material

Crystal structure: contains datablocks I, global. DOI: 10.1107/S1600536809048983/hb5232sup1.cif

Structure factors: contains datablocks I. DOI: 10.1107/S1600536809048983/hb5232Isup2.hkl

Additional supplementary materials:  crystallographic information; 3D view; checkCIF report

## Figures and Tables

**Fig. 1.**
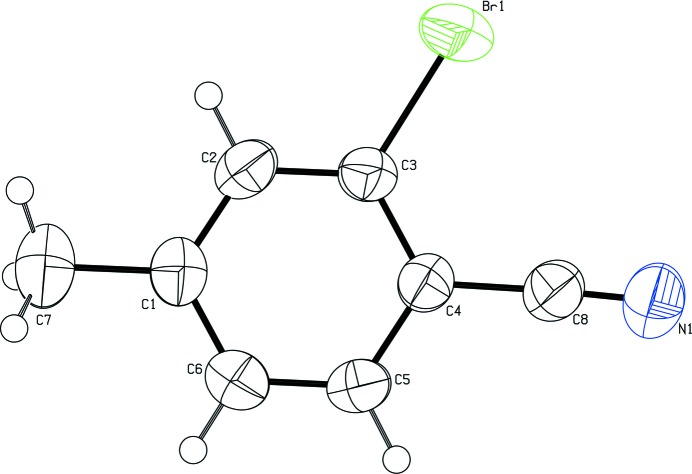
The molecular structure of (I) with 50% displacement ellipsoids.

**Fig. 2.**
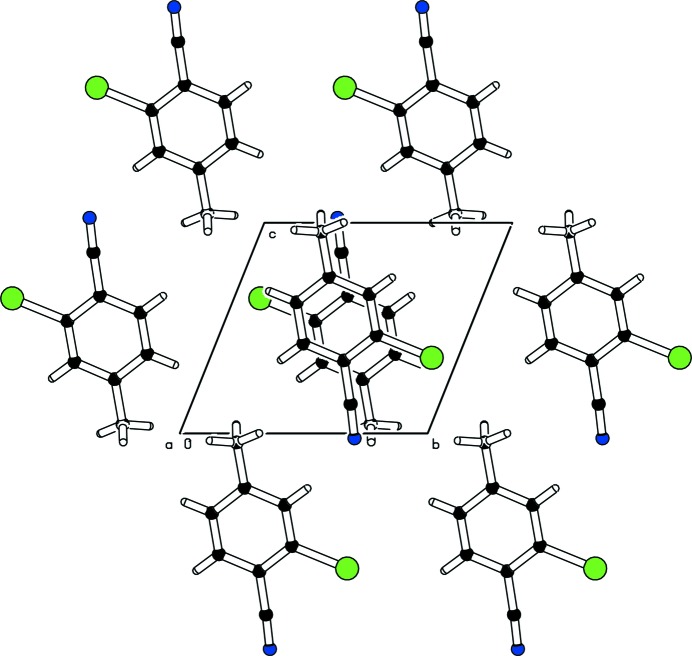
Unit cell packing diagram.

## supplementary crystallographic information

### Comment

Synthesis of 2-bromo-4-methylbenzonitrile derivatives are important compounds due to their use as intermediates in the synthesis of phthalocyanine dyes. The substituted phthalocyanine dyes have been used for photo redox reactions (Simon & Sirlin, 1989) and photodynamic cancer therapy (Simon *et al.*. 1989).

The title compound(I) is almost planar. The cyano plane (C4/C8/N1) is oriented at a dihedral angle of 79.7 (3)° with respect to aromatic ring (C1/C2/C3/C4/C5/C6). The dihedral angle between the plane containing the methyl carbon (C1/C2/C6/C7) and aromatic ring plane is 0.22 (0.18)°. No significant intermolecular or intramolecular hydrogen bonding interaction has been observed in the molecule.

### Experimental

3-Bromo-4-amino toluene (10 g, 54 mmol) (Johnson & Sandborn, 1941) was dissolved in HCl (30 ml, 17%). The mixture was cooled to 273 K in an ice-salt mixture. Over 5 min, an aqueous solution (9 ml) of NaNO_2_ (4.3 g) was added to the above mixture. The temperature was maintained at 273-278 K. A mixture of aqueous solution (6%) of Cu(I)cyanide and KCN (40%) was heated to 333 K and added to the above cold neutralized diazonium salt solution. After work up of reaction, colourless blocks of (I) were obtained by the slow evaporation of water.

### Refinement

The H atoms were geometrically placed (C—H = 0.93–0.96Å) and refined as riding with *U*_iso_(H) = 1.2*U*_eq_(C) or 1.5*U*_eq_(methyl C).

### Crystal data

**Table:**

C_8_H_6_BrN	*Z* = 2
*M**_r_* = 196.05	*F*(000) = 192
Triclinic, *P*1	*D*_x_ = 1.665 Mg m^−^^3^
Hall symbol: -P 1	Mo *K*α radiation, λ = 0.71073 Å
*a* = 7.5168 (11) Å	Cell parameters from 3073 reflections
*b* = 7.8383 (11) Å	θ = 2.2–21.2°
*c* = 7.9428 (11) Å	µ = 5.17 mm^−^^1^
α = 69.243 (7)°	*T* = 296 K
β = 64.375 (8)°	Block, colourless
γ = 87.567 (8)°	0.41 × 0.28 × 0.19 mm
*V* = 391.14 (10) Å^3^	

### Data collection

**Table:**

Bruker Kappa APEXII CCD diffractometer	1921 independent reflections
Radiation source: fine-focus sealed tube	1244 reflections with *I* > 2σ(*I*)
graphite	*R*_int_ = 0.025
φ and ω scans	θ_max_ = 28.3°, θ_min_ = 2.8°
Absorption correction: multi-scan (*SADABS*; Bruker, 2007)	*h* = −9→9
*T*_min_ = 0.226, *T*_max_ = 0.440	*k* = −10→10
8084 measured reflections	*l* = −10→10

### Refinement

**Table:**

Refinement on *F*^2^	Primary atom site location: structure-invariant direct methods
Least-squares matrix: full	Secondary atom site location: difference Fourier map
*R*[*F*^2^ > 2σ(*F*^2^)] = 0.033	Hydrogen site location: inferred from neighbouring sites
*wR*(*F*^2^) = 0.084	H-atom parameters constrained
*S* = 1.01	*w* = 1/[σ^2^(*F*_o_^2^) + (0.0326*P*)^2^ + 0.2649*P*] where *P* = (*F*_o_^2^ + 2*F*_c_^2^)/3
1921 reflections	(Δ/σ)_max_ < 0.001
92 parameters	Δρ_max_ = 0.44 e Å^−^^3^
0 restraints	Δρ_min_ = −0.49 e Å^−^^3^

### Special details

**Table:**

Geometry. All s.u.'s (except the s.u. in the dihedral angle between two l.s. planes) are estimated using the full covariance matrix. The cell s.u.'s are taken into account individually in the estimation of s.u.'s in distances, angles and torsion angles; correlations between s.u.'s in cell parameters are only used when they are defined by crystal symmetry. An approximate (isotropic) treatment of cell s.u.'s is used for estimating s.u.'s involving l.s. planes.
Refinement. Refinement of *F*^2^ against ALL reflections. The weighted *R*-factor *wR* and goodness of fit *S* are based on *F*^2^, conventional *R*-factors *R* are based on *F*, with *F* set to zero for negative *F*^2^. The threshold expression of *F*^2^ > 2σ(*F*^2^) is used only for calculating *R*-factors(gt) *etc*. and is not relevant to the choice of reflections for refinement. *R*-factors based on *F*^2^ are statistically about twice as large as those based on *F*, and *R*- factors based on ALL data will be even larger.

### Fractional atomic coordinates and isotropic or equivalent isotropic displacement parameters (Å^2^)

**Table:**

	*x*	*y*	*z*	*U*_iso_*/*U*_eq_	
Br1	0.32613 (7)	0.89370 (4)	0.35907 (6)	0.07874 (19)	
C1	0.2019 (4)	0.3498 (4)	0.7427 (4)	0.0525 (7)	
C2	0.2395 (4)	0.5395 (4)	0.6606 (4)	0.0512 (7)	
H2	0.2419	0.6033	0.7387	0.061*	
C3	0.2732 (4)	0.6352 (4)	0.4660 (4)	0.0457 (6)	
C4	0.2711 (4)	0.5439 (4)	0.3461 (4)	0.0445 (6)	
C5	0.2349 (5)	0.3537 (4)	0.4270 (5)	0.0532 (7)	
H5	0.2337	0.2899	0.3485	0.064*	
C6	0.2008 (5)	0.2588 (4)	0.6222 (5)	0.0582 (8)	
H6	0.1765	0.1310	0.6747	0.070*	
C7	0.1639 (6)	0.2446 (5)	0.9569 (5)	0.0764 (10)	
H7A	0.2191	0.3191	1.0006	0.115*	
H7B	0.2253	0.1339	0.9654	0.115*	
H7C	0.0231	0.2136	1.0415	0.115*	
C8	0.3088 (5)	0.6413 (4)	0.1400 (5)	0.0540 (7)	
N1	0.3389 (5)	0.7126 (4)	−0.0230 (5)	0.0771 (9)	

### Atomic displacement parameters (Å^2^)

**Table:**

	*U*^11^	*U*^22^	*U*^33^	*U*^12^	*U*^13^	*U*^23^
Br1	0.1226 (4)	0.0413 (2)	0.0893 (3)	0.01752 (18)	−0.0609 (3)	−0.02676 (18)
C1	0.0514 (18)	0.0578 (18)	0.0451 (17)	0.0061 (14)	−0.0231 (14)	−0.0136 (14)
C2	0.0571 (18)	0.0587 (18)	0.0515 (18)	0.0159 (14)	−0.0288 (15)	−0.0308 (15)
C3	0.0525 (17)	0.0403 (14)	0.0491 (17)	0.0096 (12)	−0.0251 (14)	−0.0193 (13)
C4	0.0449 (16)	0.0480 (16)	0.0410 (16)	0.0047 (12)	−0.0188 (13)	−0.0174 (13)
C5	0.0641 (19)	0.0478 (16)	0.0525 (18)	0.0029 (14)	−0.0249 (15)	−0.0249 (14)
C6	0.069 (2)	0.0437 (16)	0.056 (2)	0.0005 (14)	−0.0258 (16)	−0.0151 (15)
C7	0.086 (3)	0.085 (3)	0.050 (2)	0.007 (2)	−0.0321 (19)	−0.0132 (18)
C8	0.0586 (19)	0.0560 (18)	0.0478 (19)	−0.0003 (14)	−0.0225 (15)	−0.0205 (15)
N1	0.098 (2)	0.077 (2)	0.0512 (18)	−0.0056 (17)	−0.0332 (17)	−0.0171 (16)

### Geometric parameters (Å, °)

**Table:**

Br1—C3	1.882 (3)	C4—C8	1.440 (4)
C1—C2	1.380 (4)	C5—C6	1.371 (4)
C1—C6	1.384 (4)	C5—H5	0.9300
C1—C7	1.503 (4)	C6—H6	0.9300
C2—C3	1.368 (4)	C7—H7A	0.9600
C2—H2	0.9300	C7—H7B	0.9600
C3—C4	1.384 (4)	C7—H7C	0.9600
C4—C5	1.383 (4)	C8—N1	1.133 (4)
			
C2—C1—C6	118.2 (3)	C6—C5—H5	119.9
C2—C1—C7	121.0 (3)	C4—C5—H5	119.9
C6—C1—C7	120.8 (3)	C5—C6—C1	121.2 (3)
C3—C2—C1	121.0 (3)	C5—C6—H6	119.4
C3—C2—H2	119.5	C1—C6—H6	119.4
C1—C2—H2	119.5	C1—C7—H7A	109.5
C2—C3—C4	120.8 (3)	C1—C7—H7B	109.5
C2—C3—Br1	119.6 (2)	H7A—C7—H7B	109.5
C4—C3—Br1	119.6 (2)	C1—C7—H7C	109.5
C5—C4—C3	118.6 (3)	H7A—C7—H7C	109.5
C5—C4—C8	119.6 (3)	H7B—C7—H7C	109.5
C3—C4—C8	121.8 (3)	N1—C8—C4	177.7 (3)
C6—C5—C4	120.3 (3)		
